# Novel InGaSb/AlP Quantum Dots for Non-Volatile Memories

**DOI:** 10.3390/nano12213794

**Published:** 2022-10-27

**Authors:** Demid S. Abramkin, Victor V. Atuchin

**Affiliations:** 1 Laboratory of Molecular Beam Epitaxy of III–V Semiconductor Compounds, Institute of Semiconductor Physics, SB RAS, 630090 Novosibirsk, Russia; 2 Department of Physics, Novosibirsk State University, 630090 Novosibirsk, Russia; 3 Laboratory of Optical Materials and Structures, Institute of Semiconductor Physics, SB RAS, 630090 Novosibirsk, Russia; 4 Research and Development Department, Kemerovo State University, 650000 Kemerovo, Russia; 5 Department of Industrial Machinery Design, Novosibirsk State Technical University, 630073 Novosibirsk, Russia; 6 R&D Center “Advanced Electronic Technologies”, Tomsk State University, 634034 Tomsk, Russia

**Keywords:** QD-Flash, self-assembled quantum dots, quantum dots memories, non-volatile memories, universal memories, hole localization, quaternary alloy, strain

## Abstract

Non-volatile memories based on the flash architecture with self-assembled III–V quantum dots (SAQDs) used as a floating gate are one of the prospective directions for universal memories. The central goal of this field is the search for a novel SAQD with hole localization energy (*E*_loc_) sufficient for a long charge storage (10 years). In the present work, the hole states’ energy spectrum in novel InGaSb/AlP SAQDs was analyzed theoretically with a focus on its possible application in non-volatile memories. Material intermixing and formation of strained SAQDs from a Ga*_x_*Al_1−*x*_Sb*_y_*P_1−*y*_, In*_x_*Al_1−*x*_Sb*_y_*P_1−*y*_ or an In*_x_*Ga_1−*x*_Sb*_y_*P_1−*y*_ alloy were taken into account. Critical sizes of SAQDs, with respect to the introduction of misfit dislocation as a function of alloy composition, were estimated using the force-balancing model. A variation in SAQDs’ composition together with dot sizes allowed us to find that the optimal configuration for the non-volatile memory application is GaSbP/AlP SAQDs with the 0.55–0.65 Sb fraction and a height of 4–4.5 nm, providing the *E*_loc_ value of 1.35–1.50 eV. Additionally, the hole energy spectra in unstrained InSb/AlP and GaSb/AlP SAQDs were calculated. *E*_loc_ values up to 1.65–1.70 eV were predicted, and that makes unstrained InGaSb/AlP SAQDs a prospective object for the non-volatile memory application.

## 1. Introduction

**Figure 1:**
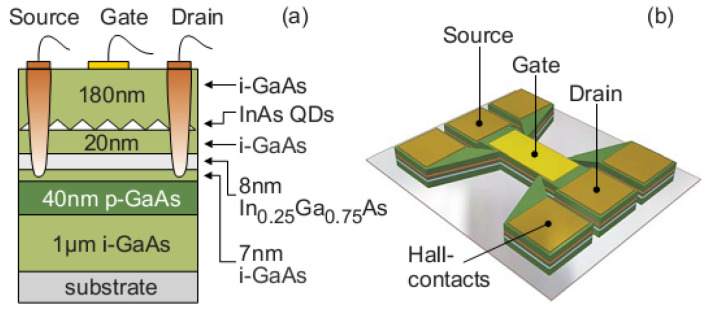
(**a**) Schematic cross section of the layered structure. (**b**) Sketch of the SAQD–Flash prototype. Hall-contacts were used for the transport measurements of 2DHG [12].

Semiconductor self-assembled quantum dots (SAQDs) are nanocrystals of a narrow bandgap material grown into a matrix or a shell of a wider bandgap material. In contrast to colloidal SAQDs [1,2], the epitaxial SAQDs formation occurs in Stranski–Krastanov growth mode as a result of surface relief reorganization [3]. One of the crucial advantages of this self-organized growth mode is the formation of a nanoscale objects array without using nanoscale lithography. The flexibility of the SAQD energy spectrum, caused by quantum confinement effects and the alloy composition variation, makes them widely applicable in broad areas of modern electronics and optoelectronics [4,5,6,7,8,9,10,11]. The energy bands offset at an SAQD/matrix heterointerface provide a charge carrier localization into SAQDs, and that opens up the prospective of using SAQDs for charge storage in non-volatile memory cells. The most interesting object in this research field is the construction of a flash memory with a III–V SAQD array used as a floating gate. The basic principles of such memory cells were formulated in [12,13]. The basic idea consists in the hole localization in the SAQD potential that permits controlling the underlying channel conductivity by the field effect, as shown in the schematic diagram presented in Figure 1 [12].

**Figure 2:**
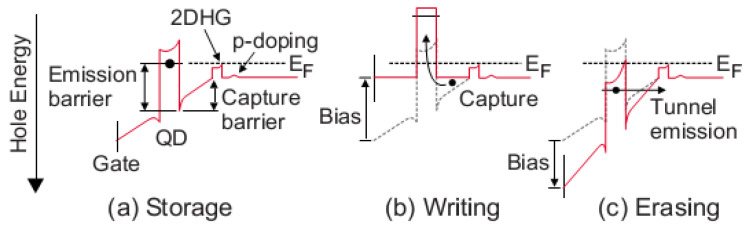
Schematic illustration of the (**a**) storage, (**b**) write and (**c**) erase operations in the SAQD–Flash concept [12].

The fabrication of the first high-temperature SAQD flash memory prototype, based on InAs/AlGaAs SAQDs [13,14], reveals the main advantages of III–V SAQD flash cells in comparison with traditional Si/SiO_2_-based ones. First of all, a significantly faster access time (about 20 ns) was mentioned [13], and that is comparable to a dynamic random-access memory (DRAM), in contrast to microsecond times common for a Si/SiO_2_-based flash memory in the framework of planar technology. As is clearly seen in Figure 2 [12], the fast access is caused by (i) the direct hole capture into an SAQD at the writing mode with the rate limited by the hole energy relaxation and recharging of the structure capacitance (*f*_cutoff_ ~ 1/(*RC)*) only and (ii) the tunnel hole emission at the erase mode [13,15]. Miniaturization of the cell III–V of the SAQD-memory will minimize the limitation of the write/erase rate caused by recharging the capacitance of the structure and allow it to approach the limit determined by the hole capture time in the SAQD (less than 1 ps [16]). Second, in traditional Si/SiO_2_-based flash memories, hot charge-carriers are used for the write/erase procedure, and it leads to a damaging of the structure and low endurance (<10^6^ cycles). The direct hole capture into SAQDs, according to the SAQD flash concept, will solve this problem and allow a significant increase in endurance. Additionally, the advantage of using III–V materials is that III–V FETs are noticeably superior to Si FETs in their speed characteristics [17]. This allows us to hope for an increase in the speed of reading data from the memory cells due to the higher mobility of charge carriers in the transistor channel. In addition, the similarity of the structure of materials III–V and Si, as well as close values of technological norms of lithography and other post-growth processes for Si and III–V chips [18], suggest that: (i) the planar cell density of III–V SAQD-memory can reach that of planar Si flash and DRAM, and (ii) III–V flash technology can walk the path that Si flash technology has traveled and take full advantage of the 3D memory architecture. All these SAQD flash memory features are a perspective for the creation of universal memory, which combines the advantages of a fast DRAM memory and a long storage time of a non-volatile flash memory, and that allows for expecting a revolution in computer architecture. It is also necessary to note that some of the III–V materials are characterized by a high probability of optical interband transitions. This allows us to hope for the development of memory with optical access, which will accelerate the transition to computing systems based on the principles of photonics.

Nevertheless, developing an SAQD-based non-volatile memory is still far from being finished. The main problem arising in this way is the insufficient hole localization energy in known SAQDs (*E*_loc_) resulting in a low charge storage time. Actually, the first high-temperature prototype based on InAs/AlGaAs SAQDs [13,14] was characterized by a storage time of about few milliseconds, caused by *E*_loc_ lower than 0.8 eV. Thus, it became clear that the main weak point of this technology is the insufficient hole localization energy in presently available III–V SAQDs. Further investigations were focused on the SAQD formation in III–V heterosystems with a higher *E*_loc_ value. This trend has involved such well-known SAQDs system as GaSb/GaAs [19,20,21] and relatively novel heterosystems, for instance, GaSb/AlGaAs [22,23], GaSb/AlAs [24], InSb/AlAs [25], InGaAs/GaP [26,27], GaSb/GaP [28,29], InGaSb/GaP [30,31] and others, as discussed in [32]. The highest *E*_loc_ value was obtained for GaSbP/GaP SAQDs (about 1.18 eV [29]), and it results in a storage time of about four days. As is mentioned in Ref. [32], a storage time longer than 10 years may be reached at *E*_loc_ > 1.3–1.5 eV.

Despite the impressively long charge storage in GaSbP/GaP SAQDs, this is not yet sufficient for a non-volatile memory cell. As it appears, a further increase in *E*_loc_ can be realized by increasing SAQD sizes and/or by the increase in the Sb content in ternary alloy GaSbP. However, it inevitably leads to a rise in strain level and a risk of plastic SAQD relaxation. On the other hand, it is possible to increase the hole localization by downshifting the matrix valence band top using AlP instead of GaP. Indeed, the valence band top in AlP lies ~0.5 eV lower than the GaP valence band top [33]. According to the simplest estimations, this allows us to expect an increase in the localization energy by the same 0.5 eV. According to calculations [12,13,32], an increase in the localization energy by 50 meV provides an increase in the charge storage time at room temperature by one order of magnitude. That is, *ceteris paribus*, replacing the matrix material from GaP to AlP can increase the storage time by 10 orders of magnitude, which corresponds to 10^8^ years. However, despite such optimistic forecasts, the InGaSb/AlP heterosystem remains unexplored either theoretically or experimentally. Earlier, the Sb-based SAQDs in the AlP matrix were just briefly mentioned in [29,32,34], and no detailed SAQD energy spectrum calculations and, especially, attempts of epitaxial growth can be found in the literature. Embedding AlGaP barriers in the heterostructures with InGaSb/GaP SAQDs [31] and AlP barriers growing close to InGaAs/GaP SAQDs [27] were only discussed.

In this contribution, we report on the InGaSb/AlP SAQDs’ energy spectrum calculations, taking into account the material intermixing and SAQD formation from the alloy with Al and P atoms. It is shown that strained GaSbP/AlP SAQDs are optimal for the non-volatile memory fabrication and allow for expecting the hole localization energy (up to 2.04 eV) along with the minimal elastic strain. Additionally, the unstrained InGaSb/AlP SAQD hole energy spectrum was investigated. The hole localization energy values are predicted up to *E*_loc_ = 1.65–1.70 eV, and it precipitates the prospects of using unstrained InGaSb/AlP SAQDs for non-volatile memory applications.

## 2. Calculation Procedure

As was mentioned, a significant variation in the SAQD energy spectrum, provided by a strong influence of SAQD geometry and alloy composition on the energy level position, is the key feature inducing the technological interest in SAQD heterostructures. On the other hand, the energy spectrum’s sensitivity to SAQD parameters makes the spectrum prediction a complicated task, requiring a wide range of parameters’ variation. The present work is aimed at the theoretical investigation of the energy spectrum for novel InGaSb/AlP SAQDs depending on SAQD sizes and the alloy composition with the focus on hole localization energy.

**Figure 3:**
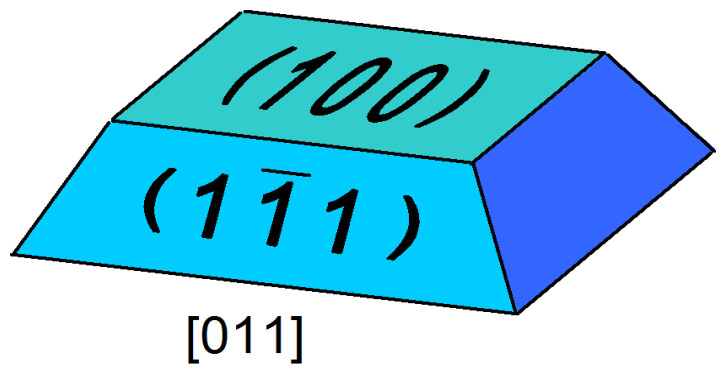
SAQD shape with the quadratic base along [011]-like directions and limited by (100) top/base planes and (111)-like side planes.

First, we need to discuss the SAQD shape, which was used for modeling. Here, it is necessary to observe the available experimental data related to the III–V SAQD formation. InAs/GaAs is the most investigated III–V heterosystem. There is a huge quantity of experimental data indicating that, for the InAs/GaAs SAQDs, the nanostructures of different shapes, including disks, truncated cones, lenses, pyramids or truncated pyramids, can be produced by Stranski–Krastanov growth techniques [6,35,36,37,38,39,40]. Comparatively, SAQDs formed in the III-phosphide matrixes were rarely studied. The experimental data related to InGaAs/GaP [26], GaSb/GaP [41] and InGaSb/GaP [30] SAQDs allow us to use the truncated pyramid shape for modeling InGaSb/AlP SAQDs. Following [41], where the dot shapes were discussed in detail, we select a pyramid with a square base oriented along [011]-like directions and limited by lateral (111) and top (100) facets with the SAQD aspect ratio of 1:4. The SAQD shape used for modeling is shown in Figure 3.

Besides the shape factor, the SAQD energy spectrum may be affected by elastic deformation. All III-phosphides and III-antimonides are of the same crystal structure (zinc blend) type, but the crystals are characterized by quite different lattice constants. In the most strained case of the InSb/AlP pair, the difference is as high as 15% [33]. Therefore, the nanoislands are inevitably formed with elastic strains. According to the model-solid theory [42], a strain leads to an energy band shift that, consequently, has its effect on the SAQD energy spectrum. Unfortunately, when the elastic energy of SAQD exceeds some critical level, plastic relaxation processes can occur and threading dislocations can be generated [43,44,45,46]. A distortion of translation symmetry in the dislocation core results in the appearance of deep centers close to the SAQDs, and it could lead to the tunnel hole escape from an SAQD and, consequently, to a dramatic shortening of hole storage time. Moreover, threading dislocations provide an electric bypass in the heterostructure that hinders the band-bending control by the gate bias. Thus, the calculation of critical SAQD sizes, accounting for the effects related to the introduction of dislocations, is an important task in our modeling.

Furthermore, material intermixing and SAQDs formation from the solid alloy result in the change in basic material parameters, such as lattice constant, bandgap and valence band offset [33], that also influence the strain value and energy band positions. The In*_x_*Ga_1−*x*_Sb deposition on AlP can, in general, result in the In*_x_*Ga_1−*x*_Sb*_y_*P_1−*y*_/AlP SAQDs formation. The material intermixing can occur during the III-Sb deposition stage due to bulk interdiffusion [47,48,49,50], as well as during the growth of cap AlP layers due to the material segregation and exchange reaction between the SAQD and the AlP matrix [51,52,53,54,55,56,57,58,59]. In order to simplify calculations, we considered SAQDs consisting of quaternary alloys Ga*_x_*Al_1−*x*_Sb*_y_*P_1−*y*_, In*_x_*Al_1−*x*_Sb*_y_*P_1−*y*_ and In*_x_*Ga_1−*x*_Sb*_y_*P_1−*y*_. As is shown below, this simplification does not decrease the interest in our results, but allows us to demonstrate the main regularity of the SAQD energy spectrum depending on the alloy composition. Thus, the calculation procedure can be subdivided into the following stages: (i) calculation of critical SAQD size depending on the SAQD alloy composition, (ii) taking into account the effect of material mixing on the SAQD material parameters, (iii) calculations of strain distribution in SAQDs, (iv) SAQD band alignment calculations and (v) charge carrier energy level calculations. Let us describe this algorithm in detail.

### 2.1. Critical Sizes Calculation

**Figure 4:**
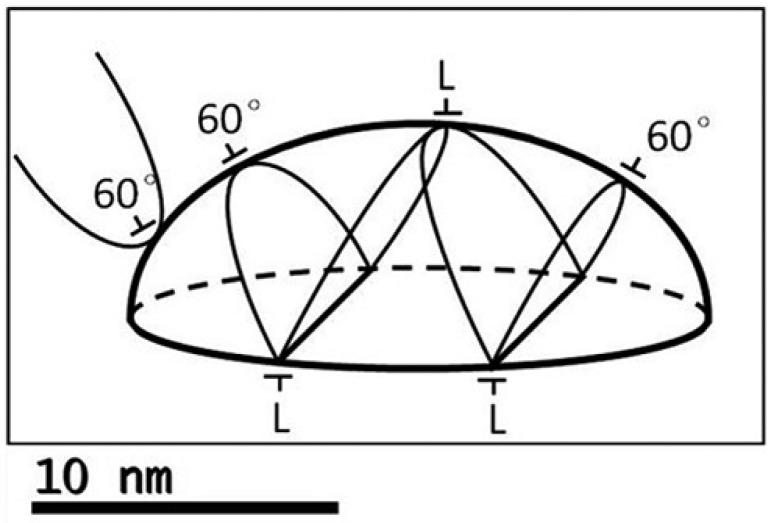
Location of dislocation loops around the SAQD; 60° dislocations tend to combine into Lomer dislocations (L). A dislocation loop (left) fails to wrap around the dot and threads towards the surface [60].

The introduction of the dislocation occurs when the critical SAQD elastic energy level is reached [43,44,45,46], and it is governed by SAQD sizes and SAQD/matrix lattice constant mismatch *f* dependent on the SAQD alloy composition. Thus, we need to calculate the critical sizes of SAQD as a function of the alloy composition. The force-balance approach [60,61] was used for the critical SAQD sizes calculation. This approach was developed by Fischer for the prediction of critical thickness when the dislocations are introduced in strained films, and, also, it was successfully used for the critical thickness prediction in the GeSi/Si layers [61]. For the III–V SAQD, the approach was first used in [60]. In the framework of this approach, the equilibrium situation is provided by the competition of two forces: (i) strain-assisted force that leads to the dislocation introduction and (ii) dislocation loop tension directed to the minimization of the total dislocation length. To simplify the calculations, in our case, the problem was simplified to the interaction between two 90° Lomer dislocations. Indeed, as is presented by the SAQD sketch given in Figure 4 [60], 60° dislocations in the SAQD can join and form 90° Lomer dislocations. These Lomer dislocations, lying at the bottom and top SAQD heterointerfaces, form the dislocation dipole (i.e., dislocations with equal, but opposite Burgers vectors).

A distance between dislocations in a dipole corresponding to a dot height can be obtained from the equilibrium condition on the forces acting on dislocation loops, and it leads to the absence of excess shear stress *τ*_exc_:

(1) $$\tau_{exc}=\cos\lambda \;\cos\varphi \;\frac{2G\left(1+\nu \right)}{1-\nu }\left(f-\frac{b\;\;\cos\lambda }{2R_{h,p}}\left(1+\beta \right)\right)=0$$

with

(2) β=1−ν44πcos2λ  1+νlnRh,pb

(3) Rh,p=4h2+4p2−12

where *λ* = 60° is the angle between the Burgers vector and the direction in the interface plane that is normal to the dislocation line, *φ* = 35.3° is the angle between the slip plane and the strained interface normal, *G* is the shear modulus, *ν =* 0.31 is the Poisson ratio (close for all binary antimonides and phosphides), *f* is the lattice mismatch between an SAQD alloy and AlP matrix, *b =*$a/\sqrt{2}$ is the Burgers vector magnitude (*a* is the SAQD lattice constant), *h* is the separation between two segments of the dislocation dipole and *p* is the lateral separation between two dislocations. Since the introduction of one dislocation was considered, the *p* value was increased to the infinity. Solving this transcendent equation by the graphical method (see sample plot in Figure S1 in the Supplementary Materials) and varying *f* and *b* according to the alloy composition by the linear Vegard law for quaternary alloys of generalized composition *A_x_B*_1−*x*_*C_y_D*_1−*y*_ ($a_{ABCD}=xy\;\cdot a_{AC}+\;(1-x)y\cdot a_{BC}+\;(1-x)(1-y)\cdot a_{BD}+x(1-y)\;\cdot a_{AD}$), we obtained the critical SAQD height (*h*_c_) as a function of alloy composition. The obtained *h*_c_ values were used for consequent calculations.

### 2.2. Effect of Alloy Composition

The formation of a solid alloy from different atom species results in a disorder and material parameter fluctuation on a micro scale, comparable with few interatomic distances [62,63,64,65]. However, typical SAQD sizes of several nanometers [3,4,5,6,7,8,9,10,11] significantly exceed this distance, and this fact allows us to neglect the disorder and consider a solid alloy as a material with averaged constant parameters governed by the alloy composition. Material parameters for quaternary alloys of the *A_x_B*_1−*x*_*C_y_D*_1−*y*_ type were calculated in the quadratic approach using expression [33]:

(4) $$\begin{array}{l} P_{ABCD}=xy\cdot P_{AC}+\;(1-x)y\cdot P_{BC}+\;(1-x)(1-y)\cdot P_{BD}+x(1-y)\;\cdot P_{AD}+\\ +x(1-x)y\cdot \;C_{ABC}+\;(1-x)y(1-y)\cdot \;C_{BCD}+x(1-x)(1-y)\cdot \;C_{ABD}+xy(1-y)\cdot {\;C}_{\mathrm{ACD}} \end{array}$$

where *x* and *y* are the fractions of corresponding atoms, *P*_ij_ is the parameter value for binary compounds and *C*_ijk_ is the bowing parameters for the corresponding ternary alloys.

During the SAQD formation, material intermixing processes may result in the distribution of alloy composition over the SAQD bulk. The studies of composition variation in the well-known ternary alloy In*_x_*Ga_1−*x*_As/GaAs SAQDs yielded a reverse triangle distribution of the In content along the growth axis [35,36], where the In content rises to the SAQD top. However, quaternary and more complicated alloy SAQDs have not been investigated in such detail. In one of few contributions related to the consideration of multielement alloy SAQDs, the experimental data were reported for the atom distributions in InGaAsSb/GaP SAQDs grown on the GaAs sublayer [66]. The non-uniform element distributions along the growth direction were determined. The trends of element distribution were close to the triangle shapes, but the peak positions for In and Sb were shifted to the SAQD top in reference to the peak positions for Ga, P and As, and that was caused by drastically different segregation effects. However, the available data are not essential to see how the element profiles are changed as a function of average element content. Therefore, in order to simplify the calculation procedure, we used the SAQD model with a fixed alloy composition over the SAQD bulk.

### 2.3. Strain Distribution

**Figure 5:**
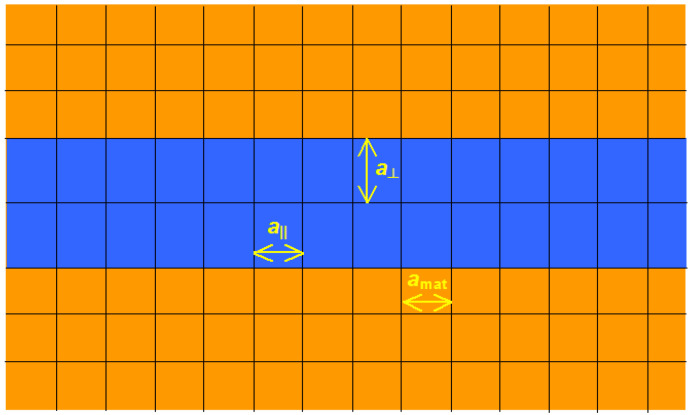
Thin layer of the material with a high lattice constant coherently strained to the matrix of a material with a low lattice constant.

Since the typical SAQD size drastically exceeds the interplanar distances in III–V materials, the SAQD material can be considered as a continuous matter. The strain was calculated in the framework of the model-solid approach [42]. A thin 2D layer coherently strained to the surrounding matrix and arranged along the (100) plane is schematically presented in Figure 5. Both layer and matrix materials are related to the zinc blend type. The lateral lattice constant of the thin layer is equal to the lattice constant of the unstrained matrix:

(5) $$a_{\left|\right|}^{}=a_{mat}^{}$$

Therefore, the lateral component of deformation tensor *ε_xx_* can be written as:

(6) $$\varepsilon_{xx}=\varepsilon_{yy}=\frac{a_{\left|\right|}^{}-a_{0}^{}}{a_{0}^{}}=\frac{a_{mat}^{}}{a_{0}^{}}-1=f$$

where $a_{0}^{}$ is the unstrained lattice constant of thin layer material. According to the model-solid approach, the in-plane deformation induces the corresponding deformation along the growth axis. The lattice constant of the thin layer in the growth axis direction is:

(7) $$a_{⊥}^{}=a_{0}^{}\left(1-2\frac{C_{12}}{C_{11}}\varepsilon_{xx}\right)$$

where *C*_12_ and *C*_11_ are the elastic constants of a thin layer material. Thus, the component of the deformation tensor along the growth axis is:

(8) $$\varepsilon_{zz}=\frac{a_{⊥}^{}}{a_{0}^{}}-1=-2\frac{C_{12}}{C_{11}}f$$

In this simple case, the deformation is almost localized in the thin strained layer, in contrast to the case of 3D SAQD, where the surrounding matrix layers are also strained [67,68,69,70]. The strain distribution over the SAQD volume and attached matrix material was calculated by the elastic energy minimization technique. This technique is based on the mesh point positions’ variation until the total elastic energy of the system reaches a minimum. The calculations were performed using the Nextnano++ program package [71].

### 2.4. Band Alignment Calculation

The SAQD band alignment was calculated in two steps [42]. First, the band alignment for an unstrained SAQD was obtained using the valence band offsets (VBO) and bandgap energy values for the solid alloy [33]. Then, the band edge shift due to the elastic strain was taken into account using deformation potentials [42]. The strain itself can be divided into the hydrostatic strain $H=\varepsilon_{xx}+\varepsilon_{yy}+\varepsilon_{zz}$, that controls the change in the unit cell volume, and biaxial strain $I=\varepsilon_{zz}-\varepsilon_{xx}$, that accounts for the distortion of the unit cell shape. A hydrostatic strain leads to a change in charge density in a crystal, and that has the effect on bandgaps. The biaxial strain results in the reduction in the unit cell symmetry, and it affects the degenerated energy bands’ splitting in *X* electron valleys and heavy-, light- and spin-orbital splitting hole sub-bands at the *Г* point of the Brillouin zone for the zinc blend type crystals. Note that *L* electron valleys are not subject to splitting in the case of the (100) oriented interface. The band edge shifting due to hydrostatic strain *H* can be calculated by the following simple expression:

(9) $$\Delta E_{i}^{H}=a_{i}\;H$$

where index *i* means the electron *Г*, *X* or *L* valley, or valence band and *a*_i_, is the corresponding hydrostatic deformation potential. The *X* electron band edge splits into *X_Z_* sub-bands, where the electron quasi-momentum is orthogonal to the interface, and *X_XY_* sub-band, where the electron quasi-momentum is parallel to the interface plane. The corresponding energy shifts were determined by expressions:

(10) $$\Delta E_{Z}^{}=\frac{2}{3}b_{X}\;I$$

(11) $$\Delta E_{XY}^{}=-\frac{1}{3}b_{X}\;I$$

where *b_X_* is the shear deformation potential for the *X* electron valley. Note that, for the typical case of a narrow bandgap material layer with high lattice constant embedded into a wide bandgap material matrix with low lattice constant, the value of biaxial strain *I* is positive. In combination with the positive values of *b_X_* for all III–V materials (see *Section 2.6 Materials Parameters* and *Supplementary Materials*), this fact makes Δ*E*_Z_ positive and Δ*E_XY_* negative. The energy splitting for hole subbands is described by relations:

(12) ΔElh=−16Δ0+14δE001+12Δ02+Δ0δE001+94δE001212

(13) $$\Delta E_{hh}=\frac{1}{3}\Delta_{0}-\frac{1}{2}\delta E_{001}$$

**Figure 6:**
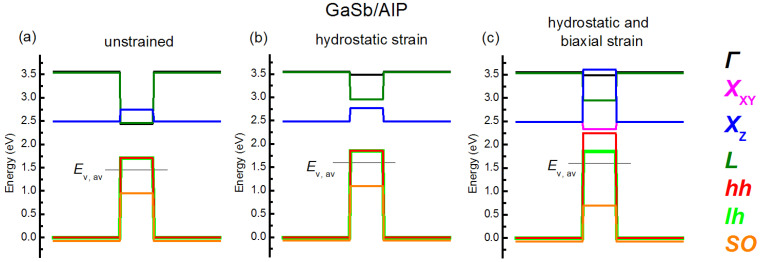
Band alignment for the thin GaSb layer coherently strained to the AlP matrix oriented along the (100) plane. The calculation is performed for 300 K. The band alignment for the unstrained GaSb/AlP layer (**a**), energy shifts due to the hydrostatic strain (**b**) and the both of the hydrostatic and the biaxial strains (**c**) are presented.

(14) ΔESO=−16Δ0+14δE001−12Δ02+Δ0δE001+94δE001212

where *δE*_001_ = 2*b_v_I*, with valence band shear deformation potential *b_v_* and Δ_0_—spin-orbit splitting. Note that, in these expressions, energy shifts were calculated in relation to the averaged valence band energy, which lies Δ_0_/3 lower than the valence band top of an unstrained zinc blende crystal. For clarity, in order to illustrate the band alignment formation for a strained thin layer, we present all the above-described stages in Figure 6 by the example of GaSb/AlP. The band diagram for the unstrained heterojunction is presented in Figure 6a. The energy shifts due to the hydrostatic strain are accounted for in Figure 6b. As is clearly seen from the figure, the hydrostatic strain leads to the shifts in electron and hole band edges without splitting. The total effect of the hydrostatic and biaxial strains on the band alignment is shown in Figure 6c. The biaxial strain leads to energy band splitting, as it is governed by Expressions (10) and (11).

### 2.5. Energy Levels

**Figure 7:**
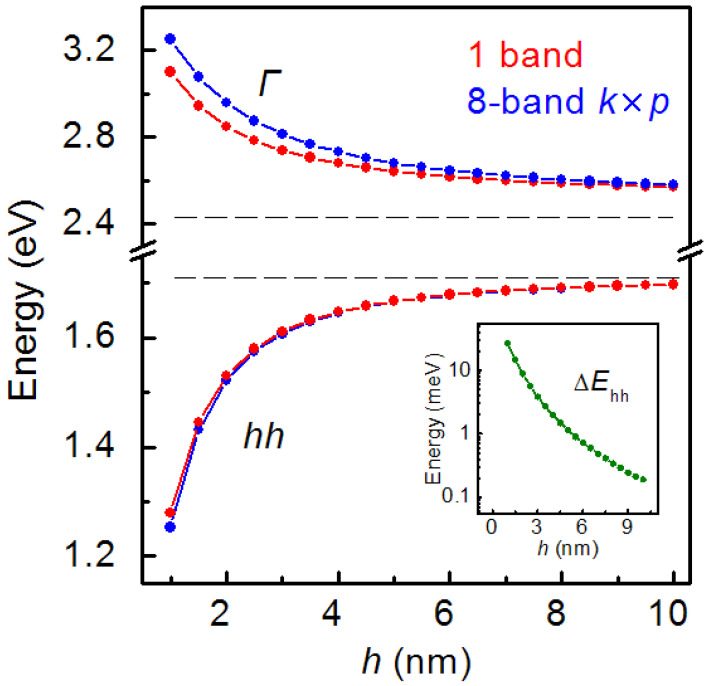
Energy levels for the ground state *Г* electrons and holes in unstrained GaSb/AlP SAQDs as a function of SAQD height. The calculation was performed in the simple band approach (red line-dots) and in the 8-band *k* **×** *p* approach (blue line-dots). The horizontal dashed lines show the band edge energies of the *Г* valley of the conduction band and the heavy hole sub-band for GaSb. The energy difference between the hole states, calculated in different approaches, is presented in the inset.

The crucial feature of SAQDs, that distinguishes them from other low-dimensional semiconductor heterostructures such as quantum wells or quantum wires, is the 3D charge-carrier localization due to small SAQD sizes, which is comparable with the de Broglie wavelengths of electrons and holes. In order to calculate the charge carrier energy levels in an SAQD, we need to solve the 3D Schrödinger equation for a potential well formed by the SAQD band alignment. The simplest way is a calculation in the simple band approach, when charge carriers are considered as quasi-particles and where the effective mass and the interband interaction is not accounted. However, the interband interaction might result in a shift of energy levels, and it may be significant. The interaction between *Г* electrons and heavy-, light- and spin-splitting holes can be taken into account in the framework of the 8-band *k* **×** *p* approach [72]. We perform the test calculation of energy levels in the unstrained GaSb/AlP SAQD for different sizes using simple band and 8-band *k* **×** *p* approaches for comparison. The obtained results are shown in Figure 7. The calculations were performed using the Nextnano++ program package. This program package is commonly used for III–V SAQD energy spectrum calculations [25,28,73,74].

We need to note that the calculations were performed taking into account *Г* electrons’ states and heavy-, light- and spin-splitting holes’ states into SAQDs, without a consideration of the *X* electrons’ states into the AlP matrix. Nevertheless, in the comparison of the energy of *Г* electrons with the *X* band edge in AlP shows, the ground electron states of unstrained GaSb/AlP SAQDs lie in the *X* valley of a conduction band of AlP. As is clearly seen from the curve behavior, the energy levels are shifted to the GaSb band edge in the SAQD, as shown in Figure 7 by dashed lines, with the increase in SAQD sizes. It is caused by reducing the quantum confinement effects. It is necessary to note that the difference in the *Г* electron energy level position, calculated in different approaches, exceeds 150 meV at the minimal SAQD height of 1 nm, and the value decreases with the dot size increase. It is topical to compare this energy difference with the uncertainty of material parameters reported for III–V compounds. The measurements of the GaSb direct bandgap at 300 K, performed by the photoreflectance and absorption spectroscopy techniques [75,76,77], yield the values in the range of 0.723–0.727 eV. Comparatively, VBO for the GaSb/AlP heterointerface is known with an accuracy of about 50 meV, as was discussed in [78]. Since this uncertainty is lower than the obtained energy difference, we conclude that, for a correct prediction of *Г* electron level position, the 8-band *k* × *p* approach is more useful. However, as can be seen in Figure 7, the energy difference between the results found by the simple band and 8-band *k* × *p* approaches for hole states is about 26 meV at the minimal SAQD height and even falls down lower than 1 meV with a dot size increase. This difference (i) does not exceed the VBO uncertainty and (ii) is not so significant in comparison with the hole localization energy, which is about 1.2–1.65 eV. This allows for the conclusion that, for the hole state energy calculation with the focus on *E*_loc_, simple band and 8-band *k* × *p* approaches yield close results. Taking into account the easier and faster calculations with the use of the simple band approach, just this model was used for the following calculations.

### 2.6. Material Parameters

The material parameters, such as lattice constants, elastic constants, VBO, bandgap energies for *Г*, *X* and *L* valleys, spin-orbit splitting energy, effective charge-carrier masses, hydrostatic and shear deformation potentials and band parameters for 8-band *k* × *p* calculations for AlP, GaP, InP, AlSb, GaSb and InSb, and corresponding bowing parameters for ternary alloys, which were used for calculations, were taken from [33,79,80,81]. All used parameters are presented in Tables S1 and S2 in the Supplementary Materials.

## 3. Results

### 3.1. Critical SAQD Sizes

**Figure 8:**
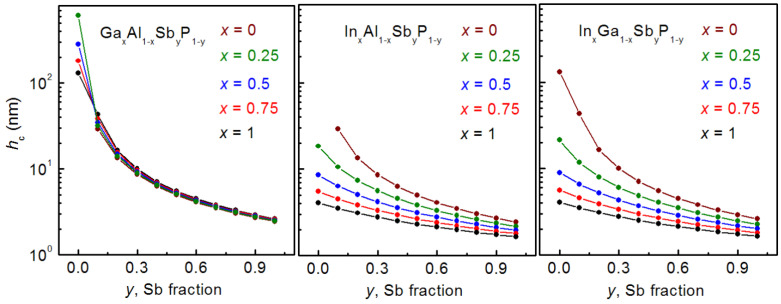
Dependences of *h*_c_ on the Sb fraction (*y*) for different contents of group III element (*x*) in Ga*_x_*Al_1−*x*_Sb*_y_*P_1−*y*_, In*_x_*Al_1−*x*_Sb*_y_*P_1−*y*_ and In*_x_*Ga_1−*x*_Sb*_y_*P_1−*y*_ SAQDs embedded in the AlP matrix.

First of all, the critical SAQD sizes calculated for different quaternary alloy compositions are presented in Figure 8.

As shown in the figure, increasing the Sb fraction in the SAQD composition leads to a drastic decrease in *h*_c_, and this is caused by the increase in related lattice constants mismatch. At the same time, *h*_c_ is practically not changed with the variation in Ga fraction in Ga*_x_*Al_1−*x*_Sb*_y_*P_1−*y*_ SAQDs because of close lattice constants in the GaP and AlP and in the GaSb and AlSb pairs [33]. On the contrary, increasing the In content in In*_x_*Al_1−*x*_Sb*_y_*P_1−*y*_ and In*_x_*Ga_1−*x*_Sb*_y_*P_1−*y*_ SAQDs results in the appreciable *h*_c_ reduction governed by the trend of lattice constants mismatch. Note that the *h*_c_ value, for the most strained InSb, GaSb and AlSb SAQDs in the AlP matrix, is about 1.6, 2.6 and 2.6 nm, respectively. It is well-known that the localization energy in the SAQDs decreases with the SAQD size reduction due to the quantum confinement effect and that E_loc_ also increases with the increase in the fraction of narrow bandgap material in the alloy composition. Based on the strong dependences of *h*_c_ on the alloy composition (*x,y*), we expect a competition between the quantum confinement effect and the alloy composition effect in the determination of the hole energy level’s position and, consequently, hole localization energy. This competition would manifest itelf in the nonmonotonic character of *E*_loc_ (*x,y*) dependencies. However, our expectations are not justified, and the *E*_loc_ (*x,y*) dependencies are monotonic in general, as will be demonstrated further.

### 3.2. Strain Distribution and Band Alignment

**Figure 9:**
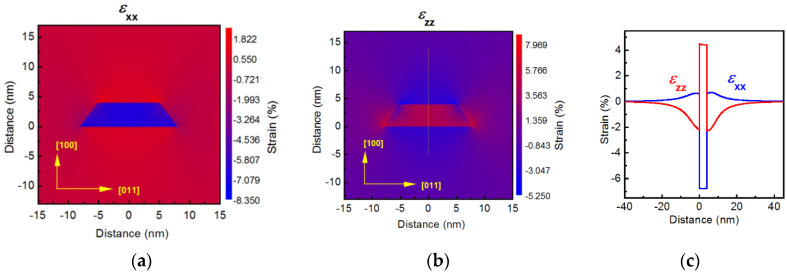
Strain distributions calculated for GaSb_0_._65_P_0_._35_/AlP SAQD with the height of 4 nm: (**a**) 2D map of *ε_xx_* component, (**b**) 2D map of *ε_zz_* component and (**c**) *ε_xx_* and *ε_zz_* trends along the central vertical SAQD axis marked by a thin yellow dashed line in panel (**b**).

The obtained critical SAQD sizes were used for the strain calculations. The calculated strain distribution in GaSb_0_._65_P_0_._35_/AlP SAQD with the height of 4 nm is presented in Figure 9. As is shown below, this alloy composition provides the minimal elastic strain along with the *E*_loc_ value sufficient for application in non-volatile memories (~1.50 eV [12,13]). As is clearly seen in Figure 9, the strain distribution is strongly heterogeneous, and the deformation is not localized in the SAQD bulk. This provides a partial strain relaxation into the SAQD and reduces absolute peak values of deformation tensor components down to *ε_xx_* = 6.78% and *ε_zz_* = 4.49%, compared to 6.83%, as governed by the lattice constants mismatch. The results are in good agreement with the III–V SAQD strains discussed in the literature [67,68,69,70]. Note that the strain distribution is not significantly changed by the alloy composition variation.

### 3.3. Band Alignment and Localization Energy

**Figure 10:**
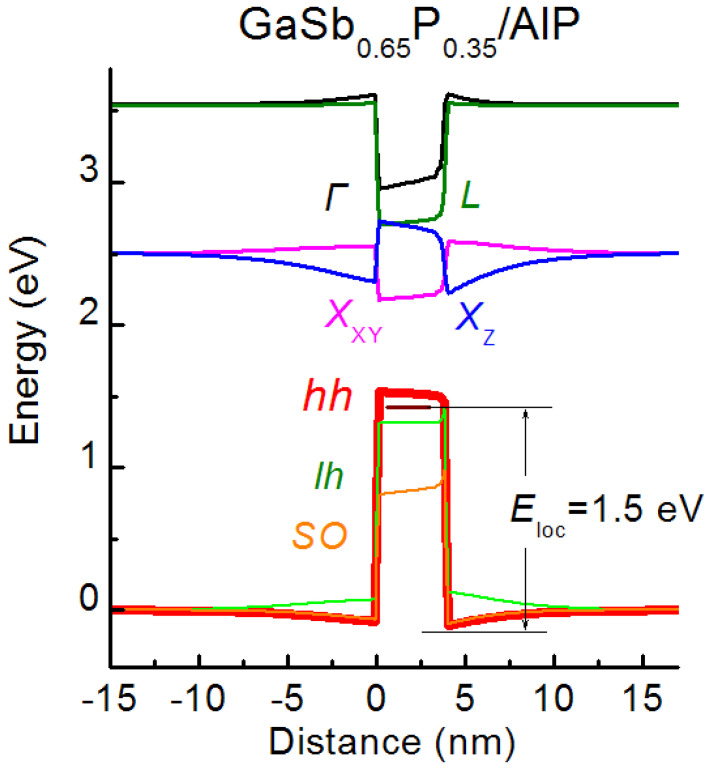
Calculated band alignment for the GaSb_0_._65_P_0_._35_/AlP SAQD.

The calculated band lineups of GaSb_0_._65_P_0_._35_/AlP SAQD are presented in Figure 10. The band edge trends along the central SAQD axis for the *Г*, *X*_z_, *X*_XY_ and *L* valleys of the conduction band and the heavy-, light- and spin-orbital splitting hole sub-bands are presented in the figure. The ground hole and electron states of the SAQD belong to the heavy hole band and the *X*_XY_ valley of conduction band, respectively, forming a band alignment of type-I. It is known that the heterostructures with the band alignment of type-I have a potential that localizes electrons and holes into the central narrow-bandgap material, while the heterostructures with the band alignment of type-II can localize only one kind of charge-carriers, electrons or holes in the central SAQD region and, for nonlocalized carriers, the potential works as a barrier [82,83]. The variation in SAQD alloy composition and size shows that the ground hole state of SAQDs lies in the subband of heavy holes, independently of the SAQD parameters. However, the composition and/or size variations can lead to a shift in the ground electron state to the *X*_Z_ valley of AlP conduction band, and it leads to the change in the type-I SAQD band alignment to type-II. It is necessary to note that the similar change in the heterostructure band alignment type was observed experimentalty for GaSb/GaP [28], GaAs/GaP [84] and InSb/AlAs [85] heterostrucrutes. Accordingly, the control of the type of heterostructure band alignment is important for the prediction of the absorption and recombination properties, which are governed by interband transitions. Since the present study is focused on the hole energy spectrum, a detailed determination of SAQD parameters controlling the band alignment type is beyond the scope of this work.

**Figure 11:**
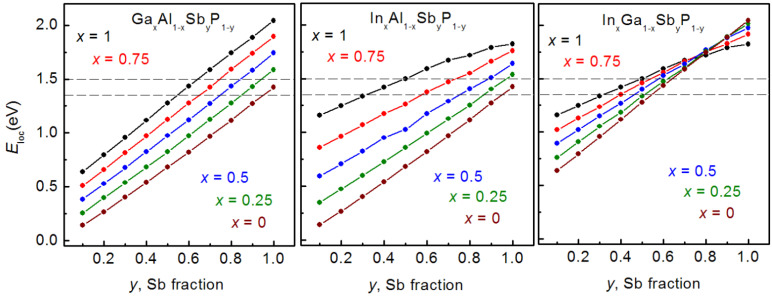
*E*_loc_ (*y*) dependences for different contents of group III elements, as calculated for Ga*_x_*Al_1−*x*_Sb*_y_*P_1−*y*_, In*_x_*Al_1−*x*_Sb*_y_*P_1−*y*_ and In*_x_*Ga_1−*x*_Sb*_y_*P_1−*y*_ SAQDs embedded in the AlP matrix. Horizontal dashed lines indicate the energy range of *E*_loc_ = 1.35–1.5 eV required for 10 years of hole storage [12,13,32,34].

The localization energy *E*_loc_ is determined as an energy difference between the ground hole state and AlP valence band edge with the minimal energy in the SAQD vicinity, as shown in Figure 10. The dependence of *E*_loc_ on the Sb fraction (*y*) in Ga*_x_*Al_1−*x*_Sb*_y_*P_1−*y*_, In*_x_*Al_1−*x*_Sb*_y_*P_1−*y*_ and In*_x_*Ga_1−*x*_Sb*_y_*P_1−*y*_ SAQDs, at several fixed Ga or In fractions (*x*), is presented in Figure 11.

As is evident from the curve observation, with the Sb fraction increase, *E*_loc_ monotonically increases in all three systems, and a value as high as ~2.04 eV is observed for pure GaSb/AlP SAQDs. The increase in Ga and In contents in Ga*_x_*Al_1−*x*_Sb*_y_*P_1−*y*_ and In*_x_*Al_1−*x*_Sb*_y_*P_1−*y*_ SAQDs, respectively, also results in the *E*_loc_ increase. These results indicate that the variation in *E*_loc_ value is mainly determined by the change in alloy composition due to the changes in VBO. Indeed, as was shown by the SAQD critical size calculations, the increase in the fraction of narrow band gap material (InSb or GaSb) in the alloy is accompanied by a reduction in *h*_c_. Nevertheless, the continuous *E*_loc_ growth with the increase in Sb or Ga/In fraction is observed in Figure 11, and it indicates a relatively weak role of the quantum confinement effect in the formation of the SAQD energy spectrum due to the high effective mass values for heavy holes. Thus, the nearly linear shape of the *E*_loc_ (*y*) dependences is governed by the alloy composition variation. This factor is dominant because the energy position of the SAQD valence band top is proportional to the alloy composition [33]. The sublinear behavior of the *E*_loc_ (*y*) functions observed for the InSb*_y_*P_1−*y*_/AlP SAQDs at *y* > 0.6 may be explained by a weak contribution of the quantum confinement effect for SAQDs with *h*_c_ < 2.5 nm. In the case of In*_x_*Ga_1−*x*_Sb*_y_*P_1−*y*_ SAQDs, the *E*_loc_ (*y*) dependence changes the character smoothly from GaSb*_y_*P_1−*y*_ to InSb*_y_*P_1−*y*_ with an *x* increase. Note that, for *y* > 0.8, *E*_loc_ is decreased, with a rise of the In content in an SAQD.

## 4. Discussion

Let us discuss the calculated results in the light of a possible application of the SAQDs under consideration in non-volatile memory cells. As was shown, SAQDs formed in the InGaSb/AlP heterosystem are characterized by a high hole localization energy up to 2.04 eV, and, accordingly, they are prospective objects for non-volatile memory cells. The variations in solid alloy compositions and sizes of SAQDs allow us to estimate the optimal SAQDs configuration.

**Figure 12:**
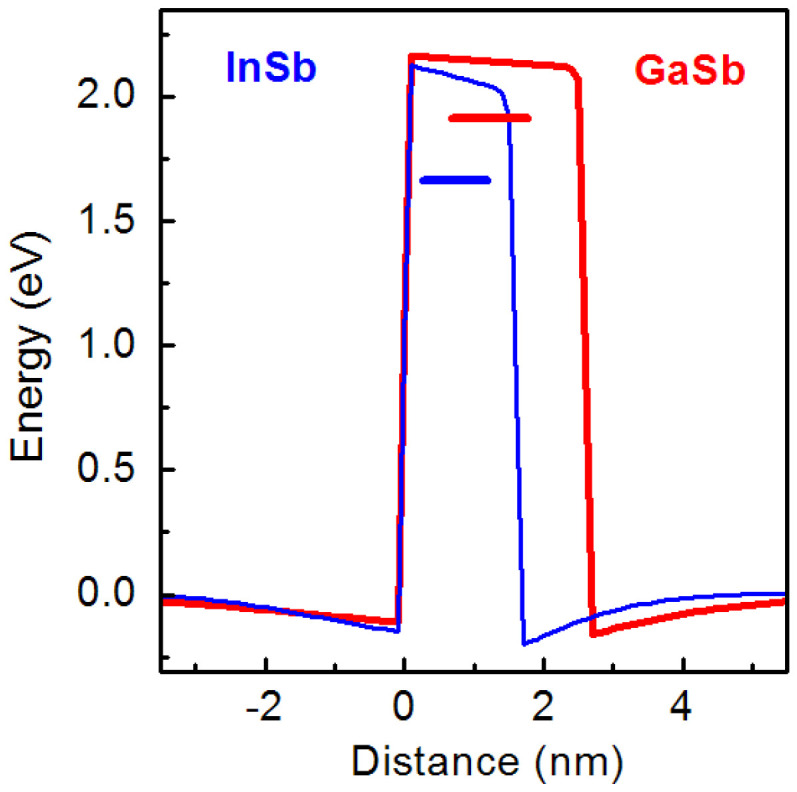
Valence band top diagrams and energy levels calculated for GaSb/AlP (red lines) and InSb/AlP (blue lines) SAQDs with critical sizes.

First of all, we need to compare two extreme cases of GaSb/AlP and InSb/AlP SAQDs. As was obtained, GaSb/AlP SAQD, with the height of 2.6 nm and base lateral size of 10.4 nm, is characterized by *E*_loc_ = 2.04 eV, while the 1.6 nm high and 6.4 nm wide InSb/AlP SAQD has *E*_loc_ = 1.82 eV. The lower localization energy in the InSb/AlP SAQD is caused by a lower valence band discontinuity and stronger quantum confinement effect, as is clearly seen in Figure 12, where the corresponding band diagrams are given. The higher *E*_loc_ value, in combination with a significantly lower lattice mismatch (10.5% in GaSb/AlP vs. 15.6% in InSb/AlP [33]), indicates that GaSb/AlP SAQDs are more attractive for the creation of non-volatile memory cells. However, from the technological point of view, it is very difficult to prepare pure GaSb SAQDs embedded into the AlP matrix due to the unavoidable material intermixing during the SAQD formation [28] and the risk of plastic relaxation of the strain [86]. Moreover, according to the predictions of the hole storage time [12,13,32,34], the value of *E*_loc_ ~ 2 eV corresponds to the giant storage times of 10^8^–10^12^ years at room temperature, depending on the hole capture cross-section. Such giant storage times (>10 years) are evidently redundant for non-volatile memories. Thus, without loss of memory functionality, an appropriate reduction in *E*_loc_ by the material intermixing is acceptable, with evident benefits of decreasing the SAQD strains. Thus, let us try to estimate the optimal SAQD configuration under the constraints of required storage time of 10 years and a minimal strain.

**Figure 13:**
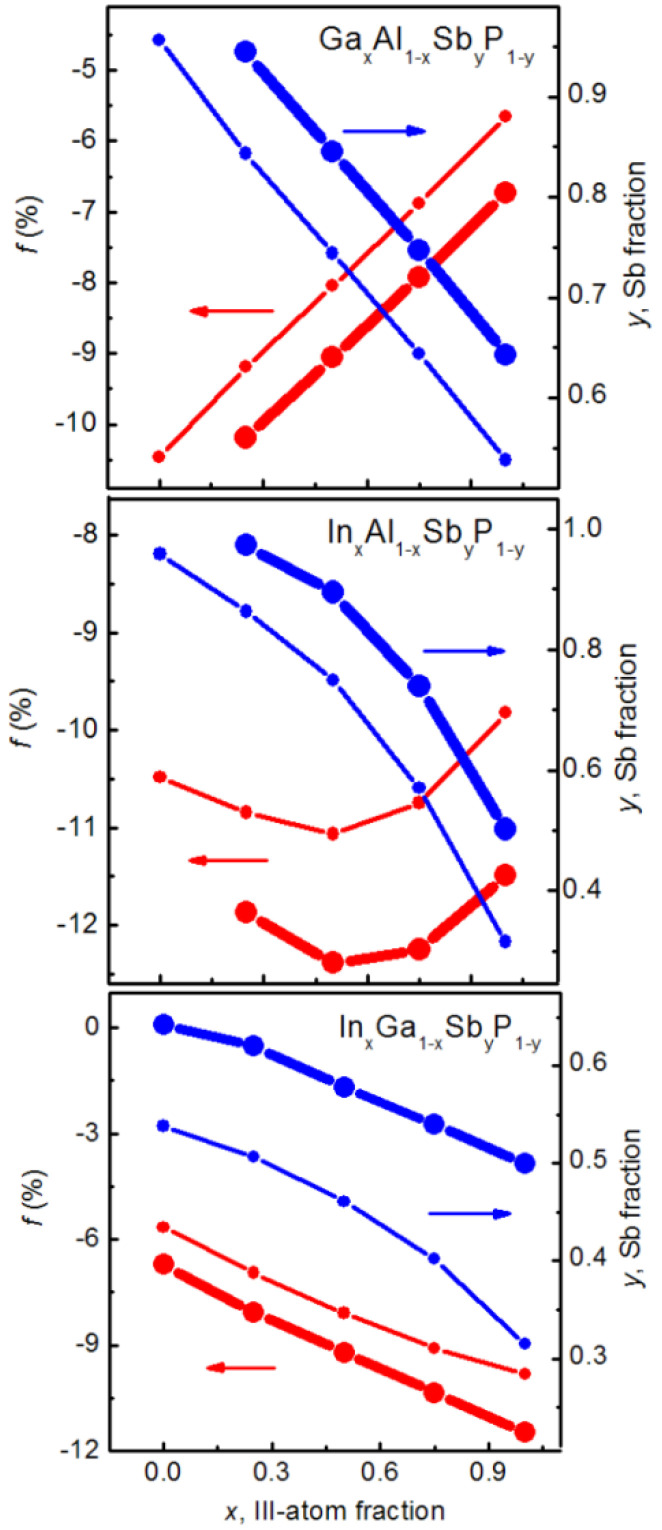
Sb fraction (*y*) as a function of group III element content (*x*) for the SAQD quaternary alloy (blue dots) calculated for the *E*_loc_ value equal to 1.35 (thin dot-lines) and 1.50 eV (thick dot-lines). Red dot-lines give the SAQD/matrix lattice constant mismatch *f* as a function of *x* with a corresponding *y* fraction.

According to [12,13], the hole storage time can be estimated by relation:

(15) ts=eEakTγT2σinf

where *T* is the temperature, *σ*_inf_ is the capture cross-section at a high temperature and γ is the coefficient independent of temperature. Localization energies of 1.35–1.50 eV are high enough for the hole storage times of ~10 years, depending on the capture cross-section *σ*_inf_ which varied in the range of 10^−12^–10^−9^ cm^2^ in GaSb/GaP [29], InGaAs/GaP [27] and InGaSb/GaP [31] SAQDs, according to the available experimental results. Since the hole capture cross-section in SAQD is determined not only by the SAQD sizes but also by the hole–phonon interaction efficiency, Auger scattering and other factors [29], the prediction of the σ_inf_ value for the SAQD with specified alloy composition and size is a complicated task. Therefore, to simplify the task, the SAQDs were considered with an *E*_loc_ value lying in the range of 1.35–1.50 eV and with the critical dot size. The possible variation in σ_inf_ was not accounted for in the calculations. In Figure 11, the horizontal dashed lines designate the targeted *E*_loc_ range. Crossing these lines with *E*_loc_ (*y*) curves determined for different *x* values spotlights a possibility to estimate the alloy composition (*x,y*) related to the targeted *E*_loc_ level. In Figure 13, the composition parameters (*x,y*) for Ga*_x_*Al_1−*x*_Sb*_y_*P_1−*y*_, In*_x_*Al_1−*x*_Sb*_y_*P_1−*y*_ and In*_x_*Ga_1−*x*_Sb*_y_*P_1−*y*_ SAQDs obtained by this algorithm are shown with blue dots.

As is seen in Figure 13, in all alloys under consideration, *y* decreases on the *x* increase. The increase in *x* from 0 to 1 induces the *y* decrease from 1 to 0.55/0.65 in Ga*_x_*Al_1−*x*_Sb*_y_*P_1−*y*_ and from 1 to 0.3/0.5 in In*_x_*Al_1−*x*_Sb*_y_*P_1−*y*_, for *E*_loc_ fixed at 1.35 or 1.50 eV, respectively. As to the In*_x_*Ga_1−*x*_Sb*_y_*P_1−*y*_ solid solution, the *x* variation from 0 to 1 results in the *y* decrease from 0.5 to 0.3 or from 0.65 to 0.5, depending on the *E*_loc_ value fixed at 1.35 or 1.50 eV, respectively. The dependences of the lattice constants mismatch *f* in the SAQD alloys and AlP matrix on the *x* value are also presented in Figure 13, and they are marked with red dot-lines. The behavior of *f* with the *x* variation in different alloys is principally different. Indeed, in Ga*_x_*Al_1−*x*_Sb*_y_*P_1−*y*_/AlP, the absolute *f* value reduces monotonically from 10.5–10% to 6–7%, depending on the *E*_loc_ value, on the *x* variation from 0 to 1. The minimal absolute *f* values correspond to GaSb*_y_*P_1−*y*_/AlP SAQDs, with *y* lying in the range of 0.55–0.65. Note that these absolute *f* values of 6–7% are close to the lattice constants mismatch in the well-known InAs/GaAs SAQDs [33]. This fact is promising for the epitaxial growth of GaSb*_y_*P_1−*y*_/AlP SAQDs because the lattice mismatch is one of the important parameters controlling the possibility of SAQD formation. The absolute lattice mismatch levels in In_x_Al_1-x_Sb_y_P_1-y_ SAQD are as high as 10–12%, and they are weakly dependent on (*x*,*y*) values, as is clear in Figure 13. This effect is in a good agreement with the higher strain in the InSb/AlP system in reference to that in the GaSb/AlP system, as was discussed above. Moreover, in In*_x_*Ga_1−*x*_Sb*_y_*P_1−*y*_ SAQD, the absolute *f* value increases up to 9.5–11.5% at *x* = 1, as shown in the bottom panel in Figure 13. Thus, the calculations allow us to point to the GaSb*_y_*P_1−*y*_/AlP SAQD with *y* = 0.55–0.65, height of 4.0–4.5 nm and a base size of about 16–18 nm as the optimal configuration with minimal strain for providing the required *E*_loc_ = 1.35–1.50 eV.

It is necessary to discuss the perspectives of the epitaxial GaSbP/AlP SAQD growth. The novel GaSb/AlP heterosystem is most similar to the more investigated GaSb/GaP system. As was mentioned in [28,29,41], the GaSb deposition on GaP results in the GaSbP/GaP SAQD formation, with the Sb fraction lying in the range of 0.3–0.7, depending on the growth conditions. This information is optimistic for the successful growth of the GaSbP/AlP SAQDs with the required Sb content. Moreover, as shown above, the optimal configuration of GaSbP/AlP SAQDs with Sb content in the range of 0.55–0.65 is characterized by the *f* level being close to the lattice mismatch observed in the well-known InAs/GaAs heterosystem, where the perfectly strained SAQDs had already been obtained. However, some difficulties in the fabrication of the GaSbP/AlP SAQDs can be assumed due to the Al-Ga intermixing during the SAQD formation.

Furthermore, the experimental data related to the formation of GaSb/GaP and GaAs/GaP SAQDs show a possibility of an exotic plastic strain relaxation mode. According to [84,86,87], at some growth conditions, a strain relaxation appeared due to the introduction of a system of 90° Lomer dislocations without 60° components. In this case, the dislocations lie at the interfaces, and they do not cross the SAQD bulk. Moreover, as was discussed in [88], the Lomer dislocation core does not contain dangling bonds, and that frees it from the deep level formation. The SAQD heterostructures, considered in [84,86,87], demonstrate a high luminescence intensity along with the radiative exciton recombination into SAQDs, and that confirms the absence of defect levels in and around SAQDs. Accordingly, it is expected that the SAQD system, where this strain relaxation mode is realized, may provide a long charge carrier storage time at appropriate *E*_loc_ values. It can be reasonably assumed that a similar strain relaxation mode can be realized in GaSb/AlP and InSb/AlP heterosystems. Thus, the calculations of *E*_loc_ for unstrained GaSb/AlP and InSb/AlP SAQDs are also interesting. Taking into account that this type of plastic relaxation leads to almost 100% relaxation of elastic deformations [84,86,87], the case of partial relaxation was not considered in this work.

**Figure 14:**
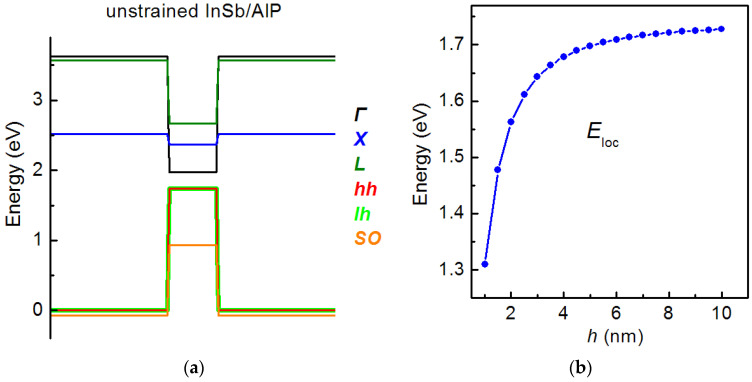
(**a**) Band alignment for the unstrained InSb/AlP SAQD. (**b**) The dependence of *E*_loc_ on the SAQD height in the unstrained InSb/AlP SAQD.

The calculations were performed for a truncated pyramid SAQD consisting of pure GaSb and InSb in the AlP matrix. Since the calculation was implemented for the unstrained SAQDs, the strain effects were not accounted for. The energy level’s position was calculated in the framework of the simple band approach. The band lineup diagram of unstrained GaSb/AlP SAQD was already provided in Figure 6a. This one, for the InSb/AlP system, is presented in Figure 14a. In the diagrams, it is clearly seen that these lineups are very similar, especially in the valence band part, and this is explained by the close values of VBO for the unstrained GaSb/AlP and InSb/AlP heterointerfaces [33]. The hole localization energy in the unstrained GaSb/AlP and InSb/AlP SAQDs lies in the range of 1.30–1.75 eV (see Figure 14b), which makes these SAQDs interesting objects for non-volatile memories, along with the strained InGaSb/AlP SAQDs.

Additionally, it should be noted that the considered InGaSb/AlP SAQDs might be attributed to the novel type of semiconductor low-dimensional heterostructures with the band alignment of type-I and indirect bandgap in some alloy compositions. These SAQDs are of interest as objects for investigations of localized exciton spin dynamics [89,90] due to the extremely long radiative lifetime of indirect excitons [91] lying in the microsecond range. As was demonstrated in [89], the fast bulk spin relaxation mechanisms are suppressed for trions in SAQDs, and this resulted in long, up to 30 μs, spin relaxation times. The coexistence of >10 years of hole storage time and microsecond exciton spin relaxation time in one structure opens the way for the creation of novel prospective hybrid memory devices based on a positive trion into SAQDs, combining a long charge storage in the floating gate with fast data processing using a trion spin.

## 5. Conclusions

The energy spectrum of novel InGaSb/AlP SAQDs was investigated theoretically with the focus on possible non-volatile memory applications. The detailed calculations of the energy spectra of strained Ga*_x_*Al_1−*x*_Sb*_y_*P_1−*y*_, In*_x_*Al_1−*x*_Sb*_y_*P_1−*y*_ and In*_x_*Ga_1−*x*_Sb*_y_*P_1−*y*_ SAQDs formed in the AlP matrix were performed for different alloy compositions and SAQD sizes. The variations were performed, keeping SAQD sizes as critical, with respect to the introduction of dislocations for different alloy compositions. It was theoretically elucidated that, among all SAQDs under consideration, the GaSbP/AlP SAQDs with the Sb fraction in the range of 0.55–0.65 have a minimal SAQD/matrix lattice constant mismatch and provided *E*_loc_ of 1.35–1.50 eV. These *E*_loc_ values are sufficient for non-volatile memory applications. This makes the GaSb/AlP heterosystem the most appropriate one for non-volatile memory cell fabrication. Additionally, the energy spectra of unstrained InSb/AlP and GaSb/AlP SAQDs were calculated. In these systems, the values of *E*_loc_ up to 1.65–1.70 eV were predicted, which also makes these SAQDs suitable for non-volatile memory applications.

## Data Availability

Data are available from the authors on request.

## Supplementary Materials

The following supporting information can be downloaded at: https://www.mdpi.com/article/10.3390/nano12213794/s1, Table S1: Materials parameters for AlP, GaP, InP, AlSb, GaSb and InSb at 300 K, which were used for the calculations. *a*_0_—lattice constant, *C*_11_, *C*_12_, *C*_44_—elastic constants, *E_g_^Г^*^,*X*,*L*^—bandgaps for *Г*, *X* and *L* valleys, *a^Г^*^,*X*,*L*^*_v_*—hydrostatic deformation potentials for the conduction band edge in *Г*, *X* and *L* points of the Brillouin zone and valence band, *b_X_*_,*v*_—shear deformation potential for the conduction band at *X* point of the Brillouin zone and valence band, Δ_0_—energy of spin-orbital splitting in the valence band, VBO—valence band offsets, *m_Г_*—electron effective mass in the *Г* point of the Brillouin zone, *m_Xt_* and *m_Xl_*—transversal and longitudinal electron effective mass in the *X* point of the Brillouin zone, *m_L_*_t_ and *m_L_*_l_—transversal and longitudinal electron effective mass in the *L* point of the Brillouin zone, *m_hh_*, *m_lh_*, *m_SO_*—effective masses for the heavy, light and spin-orbital splitting holes, *F*—Kane’s parameter, *E*_P_—Kane’s matrix element, *γ*_1,2,3_—Luttinger parameters for the valence band. Table S2: Bowing parameters for GaAlP, InGaP, AlInP, GaAlSb, InGaSb, AlInSb, AlSbP, GaSbP and InSbP [33]. Figure S1: Solving of the Equation (16) by the graphic method for the case of In0.5Ga0.5Sb0.5P0.5/AlP SAQD. Linear part of the equation depicted by the blue line, the logarithmic one by the red line. The black arrow points to the obtained hc value.
