# Supplementary material for: Novel InGaSb/AlP Quantum Dots for Non-Volatile Memories
Source: Nanomaterials (Basel). 2022 Oct 27;12(21):3794. doi: 10.3390/nano12213794 (PMC9654477; doi:10.3390/nano12213794)
Supplement: Supplementary file 1 [file nanomaterials-12-03794-s001.zip › nanomaterials-1956976-supplementary.pdf]

Supplementary Materials

Novel InGaSb/AlP quantum dots for non-volatile memories

Demid S. Abramkin[1,2], Victor V. Atuchin[3,4,5,6*]

[1]Laboratory of Molecular Beam Epitaxy of III-V Semiconductor Compounds, Institute of Semiconductor Physics, SB RAS, Novosibirsk 630090, Russia

[2]Department of Physics, Novosibirsk State University, Novosibirsk 630090, Russia

[3]Laboratory of Optical Materials and Structures, Institute of Semiconductor Physics, SB RAS, Novosibirsk 630090, Russia

[4]Research and Development Department, Kemerovo State University, Kemerovo 650000, Russia

[5]Department of Industrial Machinery Design, Novosibirsk State Technical University, Novosibirsk 630073, Russia

[6]R&D Center "Advanced Electronic Technologies", Tomsk State University, Tomsk 634034, Russia

Corresponding author: atuchin@isp.nsc.ru

Table S1. Materials parameters for AlP, GaP, InP, AlSb, GaSb and InSb at 300 K, which were used for the calculations. a_0 – lattice constant, C_{11}, C_{12}, C_{44} – elastic constants, $E_g^{\Gamma,X,L}$ – bandgaps for Γ, X and L valleys, $a_{\Gamma,X,L,v}$ – hydrostatic deformation potentials for the conduction band edge in Γ, X and L points of the Brillouin zone and valence band, $b_{X,v}$ – shear deformation potential for the conduction band at X point of the Brillouin zone and valence band, Δ_0 – energy of spin-orbital splitting in the valence band, *VBO* – valence band offsets, m_Γ – electron effective mass in the Γ point of the Brillouin zone, m_X^t and m_X^l – transversal and longitudinal electron effective mass in the X point of the Brillouin zone, m_L^t and m_L^l – transversal and longitudinal electron effective mass in the L point of the Brillouin zone, m_{hh}, m_{lh}, m_{SO} – effective masses for the heavy, light and spin-orbital splitting holes, F – Kane's parameter, E_P – Kane's matrix element, $\gamma_{1,2,3}$ – Luttinger parameters for the valence band.

Parameter	AlP	GaP	InP	AlSb	GaSb	InSb
a_0, Å	5.4672 [a]	5.4505 [a]	5.8697 [a]	6.1355 [a]	6.0959 [a]	6.4794 [a]
C_{11}, GPa	1330 [a]	1405 [a]	1011 [a]	876.9 [a]	884.2 [a]	684.7 [a]
C_{12}, GPa	630 [a]	620.3 [a]	561 [a]	434.1 [a]	402.6 [a]	373.5 [a]
C_{44}, GPa	615 [a]	703.3 [a]	456 [a]	407.6 [a]	432.2 [a]	311.1 [a]
E_g^Γ, eV	3.552 [a]	2.808 [a]	1.352 [a]	2.300 [a]	0.7267 [a]	0.173 [a]
E_g^X, eV	2.487 [a]	2.272 [a]	2.313 [a]	1.616 [a]	1.032 [a]	0.568 [a]
E_g^L, eV	3.537 [a]	2.642 [a]	1.943 [a]	1.895 [a]	0.752 [a]	0.868 [a]
a_Γ, eV	-6.88 [b]	-9.41 [b]	-6.34 [b]	-8.12 [b]	-9.33 [b]	-6.84 [b]
a_X, eV	3.98 [b]	0.63 [b]	0.59 [b]	1.91 [b]	-0.20 [b]	1.41 [b]
a_L, eV	-1.74 [b]	-4.41 [b]	-3.41 [b]	-2.91 [b]	-4.38 [b]	-2.20 [b]
a_v, eV	2.64 [b]	-0.58 [b]	-0.41 [b]	0.73 [b]	-1.32 [b]	0.31 [b]
b_X, eV	6.75 [c]	6.5 [c]	3.3 [c]	6.0 [c]	6.46 [c]	4.53 [c]
b_v, eV	-1.5 [a]	-1.6 [a]	-2.0 [a]	-1.35 [a]	-2.0 [a]	-2.0 [a]
Δ_0, eV	0.07 [a]	0.08 [a]	0.108 [a]	0.676 [a]	0.76 [a]	0.81 [a]
VBO, eV	-1.74 [a]	-1.27 [a]	-0.94 [a]	-0.41 [a]	-0.03 [a]	0 [a]
m_Γ	0.22 [a]	0.13 [a]	0.0795 [a]	0.14 [a]	0.039 [a]	0.0135 [a]
m_X^t	0.155 [a]	0.253 [a]	0.88 [a]	0.123 [a]	0.22 [a]	0.22 [e]
m_X^l	2.68 [a]	2.0 [a]	0.88 [a]	1.357 [a]	1.51 [a]	1.51 [e]
m_L^t	0.15 [d]	0.15 [a]	0.47 [a]	0.23 [a]	0.10 [a]	0.25 [a]
m_L^l	1.2 [d]	1.2 [a]	0.47 [a]	1.64 [a]	1.3 [a]	0.25 [a]
m_{hh}	0.63 [f]	0.32 [a]	0.85 [f]	0.8 [f]	0.34 [f]	0.26 [f]
m_{lh}	0.20 [f]	0.14 [f]	0.089 [f]	0.13 [f]	0.0447 [f]	0.0162 [f]
m_{SO}	0.30 [a]	0.25 [a]	0.21 [a]	0.22 [a]	0.12 [a]	0.11 [a]

F	-0.65 [a]	-	-	-	-1.63 [a]	-
E_P, eV	17.7 [a]	-	-	-	27.0 [a]	-
γ_1	3.35 [a]	-	-	-	13.4 [a]	-
γ_2	0.71 [a]	-	-	-	4.7 [a]	-
γ_3	1.23 [a]	-	-	-	6.0 [a]	-

[a][33], [b][80], [c][79], [d] GaP value, [e] GaSb value, [f] [81].

Table S2. Bowing parameters for GaAlP, InGaP, AlInP, GaAlSb, InGaSb, AlInSb, AlSbP, GaSbP and InSbP [33].

Bowing parameter	GaAlP	InGaP	AlInP	GaAlSb	InGaSb	AlInSb	AlSbP	GaSbP	InSbP
$a_{0,}$ Å	0	0	0	0	0	0	0	0	0
C_{11}, GPa	0	0	0	0	0	0	0	0	0
C_{12}, GPa	0	0	0	0	0	0	0	0	0
C_{44}, GPa	0	0	0	0	0	0	0	0	0
E_g^{Γ}, eV	0	0.65	-0.48	-0.044 +1.22x	0.415	0.43	2.7	2.7	1.9
E_g^{X}, eV	0.13	0.20	0.38	0	0.33	0	2.7	2.7	1.9
E_g^{L}, eV	0	1.03	0	0	0.4	0	2.7	2.7	1.9
a_{Γ}, eV	0	0	0	0	0	0	0	0	0
a_X, eV	0	0	0	0	0	0	0	0	0
a_L, eV	0	0	0	0	0	0	0	0	0
a_v, eV	0	0	0	0	0	0	0	0	0
b_X, eV	0	0	0	0	0	0	0	0	0
b_v, eV	0	0	0	0	0	0	0	0	0
Δ_0, eV	0	0	-0.19	0.3	0.1	0.25	0	0	0.75
VBO, eV	0	0	0	0	0	0	0	0	0
m_{Γ}	0	0.051	0.22	0	0.0092	0	0	0	0
m_X^t	0	0	0	0	0	0	0	0	0
m_X^l	0	0	0	0	0	0	0	0	0
m_L^t	0	0	0	0	0	0	0	0	0
m_L^l	0	0	0	0	0	0	0	0	0
m_{hh}	0	0	0	0	0	0	0	0	0
m_{lh}	0	0	0	0	0.011	0	0	0	0
m_{SO}	0	0	0	0	0	0	0	0	0

The critical sizes of SAQDs were obtained by solving of equation (1). It is can be presented in the following form:

$$h_c = \frac{b\cos(\lambda)}{f}\left(1+\frac{\left(1-\nu/4\right)\ln\left(\frac{h_c}{2b}\right)}{4\pi\cos^2(\lambda)(1+\nu)}\right) \quad (16).$$

In the case of the $A_xB_{1-x}C_yD_{1-y}$ quaternary alloy, lattice constant mismatch is $f = \frac{a_{mat}}{a_0} - 1 = \frac{a_{mat}}{xy\cdot a_{AC} + (1-x)y\cdot a_{BC} + (1-x)(1-y)\cdot a_{BD} + x(1-y)\cdot a_{AD}} - 1$ and Burgers vector magnitude is $b = \frac{a_{0t}}{\sqrt{2}} = \frac{xy\cdot a_{AC} + (1-x)y\cdot a_{BC} + (1-x)(1-y)\cdot a_{BD} + x(1-y)\cdot a_{AD}}{\sqrt{2}}$. We solved this equation by the graphic method, as it is illustrated in the **Figure S1.**

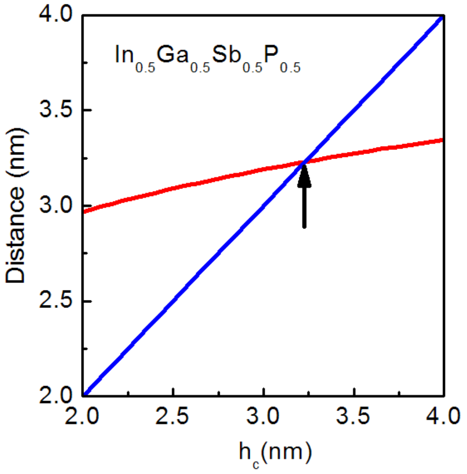

Figure S1. Solving of the equation (16) by the graphic method for the case of $In_{0.5}Ga_{0.5}Sb_{0.5}P_{0.5}$/AlP SAQD. Linear part of the equation depicted by the blue line, the logarithmic one by the red line. The black arrow points to the obtained h_c value.
